# Uterine Hemorrhage

**Published:** 1848-11

**Authors:** 


UTERINE HEMORRHAGE.—Prof. Linton, one of the editors of the *St. Louis Medical Journal,* has, in the last December number of that Journal, proposed a novel mode of treating this difficulty. It consists in the application of bandages to the legs, commencing at the foot and extending to the hip. He furnishes the particulars of a case thus treated. A lady of previous good health and robust constitution, soon after the delivery of the placenta, was seized with a profuse and alarming hemorrhage; some delay occurred in obtaining ergot, during which time a firm clot from the vagina and uterus was cast off. The state of the patient now became extremely alarming, although the uterus was tolerably firmly contracted; "the general system was *already* in a state of prostration, from which I feared it would be difficult to arouse it;" the bandages were immediately resorted to, a decided improvement in the condition of the patient was soon evinced, "and in half an hour I was able to leave her without apprehending danger." His colleague Dr. McPheeters has also witnessed its beneficial effects in a case of severe hemorrhage which occurred during a miscarriage; he states that two or three minutes after the application of the bandage the state of the pulse exhibited a decided improvement. The patients in each instance expressed themselves decidedly relieved by their use.

Dr. L., in closing the report of his case, remarks, "it is not necessary to discuss the mode in which it acts, as every one will see that it is by supplying the general system with blood from the legs." Dr. McP. says, "it is not unreasonable to suppose that six or eight ounces of blood may in this way be supplied to the general system, and to that extent the drooping powers of life sustained, while other remedies are brought to bear." Certainly their application can do no harm, and in many cases of extreme exhaustion we have no doubt but that their use would be followed by the most pleasing effects. The readiness with which they may be obtained renders it well worthy of the notice of all, and particularly those who practice in remote sections of the country. May not the difficult and dangerous practice of transfusion be at least sometimes obviated by their use?—*Ibid.*

